# Understanding the Mechanism of User Experience Role in Educational Livestreaming Platform

**DOI:** 10.3389/fpsyg.2022.907929

**Published:** 2022-05-26

**Authors:** Hong Zhao, Yajun Zhou

**Affiliations:** ^1^International College of Cultural Education, Northeast Agricultural University, Harbin, China; ^2^College of Finance and Economics, Nanchang Institute of Technology, Nanchang, China

**Keywords:** educational livestreaming platform, user experience, psychological ownership, purchase intention, privacy concerns

## Abstract

The booming development of educational livestreaming platforms has caused the prevalence of user experience to a certain extent, which profoundly affects users' purchase intention and behavior, and has become a hot topic of current research in the online education field. However, there is a lack of in-depth analysis on the mechanism of the role of user experience in influencing purchase intention. Based on the analysis of user experience and psychological ownership, this study constructs a moderated mediation model to investigate the mediating psychological mechanism and boundary conditions of user experience affecting purchase intention. In this study, a valid sample of 372 users was used for structural equation modeling analysis. The results of the study found that user experience not only had a significant positive effect on purchase intention but was also mediated by psychological ownership. We also found that the effect of psychological ownership on purchase intention was moderated by privacy concerns. This study examines the role of user experience in purchase intention and reveals the mechanism of the role of user experience in an educational livestreaming platform.

## Introduction

With the rapid development of network technology, educational livestreaming platforms have emerged in large numbers, gradually becoming a new platform for users to learn online, share knowledge, and communicate online, attracting a large number of users (Zheng et al., 2018; Guo et al., 2021). The value of educational livestream platforms is becoming more and more prominent, and has become a hot topic of marketing research in the context of the Internet. The value of educational livestreaming platforms is not only in the traditional e-commerce for the purpose of transaction but also in the rich experience that users get in the process of using an educational livestreaming platform, which can form the user's will to choose whether to buy an educational livestreaming platform or not. The authenticity of the message of the videos and copy shared in educational livestreaming platforms is very strong, which helps to enhance the user's information experience and further the transaction, etc. Yu et al. (2015) argued that as an indispensable part of the business development process, user experience deserves high attention from companies. Lu et al. (2021) showed that the better the user's educational livestreaming platform experience, the higher their willingness to continue using it. Currently, academic research on educational livestreaming platforms is more focused on the educational livestreaming platform itself and how to improve user behavior and marketing performance from a marketing perspective, while specific research on user experience in the educational livestreaming platform is still limited. How to motivate users and ensure the sustainability of the educational livestreaming platform and promote users' purchase intention are major challenges for operators of the educational livestreaming platform. Therefore, this research is an empirical study on the impact of user experience on purchase intention of the educational livestreaming platform.

In the context of educational livestreaming platform usage, users feel that the educational livestreaming platform is a state of consciousness that belongs to them, and this state of consciousness leads to a more positive value judgments and behavioral responses to the educational livestreaming platform. In other words, an individual will have a “psychological ownership” of the educational livestreaming platform, which makes the individual feel that the target or a part of the target belongs to him or her, which is essentially a “psychological sense of possession” (Pierce et al., 2001). The user's sense of “psychological possession” of the educational livestreaming platform reflects the close connection between the individual's self-concept and the educational livestreaming platform, and can even become an extension of the self, reflecting the individual's awareness, thoughts, and beliefs about the ownership of the educational livestreaming platform, along with emotional components such as pleasure and self-efficacy.

Studies have found that not only employees have psychological ownership of organizations but users also have psychological ownership of companies or products (Jussila et al., 2015; Kirk et al., 2018). Psychological ownership only has a sense of control, possession, identity, etc, over the target that is recognized and protected by the individual themselves. According to Peck and Shu (2009), Kamleitner and Feuchtl (2015), psychological ownership is a potential intermediate mechanism for the influential role of user experience in individual purchase intention and behavior, which helps to explain the role of user experience. role of influence on individual's psychology, customer satisfaction, improved customer citizenship behavior, and other outcomes. Research has shown that companies use different methods to stimulate employees' sense of efficacy, space, and self-identity to create psychological ownership, and that the positive attitudes, self-concept and sense of responsibility shown by employees' psychological ownership lead to positive behaviors toward the company (Lee and Suh, 2015). Therefore, in order to explore the intermediate mechanisms through which user experience influences their purchase behavior, this article will examine how user experience influences individual purchase intention through the potential mechanism of psychological ownership. This study will explore this issue from the perspective of both the direct effect of user experience on individual purchase intention and the indirect effect through psychological ownership.

In educational livestreaming platform context, differences in users' privacy concerns may influence the role of user experience in purchase intention. As the use of the Internet, social networks, and other forms of information sharing has become more widespread, the discussion of privacy concerns has gradually become the focus of much research (Zang and Zhou, 2020). The attitudes and behaviors of users when participating in marketing campaigns organized by the educational livestreaming platform should also take into account personal concerns about privacy breach and privacy protection (Hu and Qiu, 2018). For example, public or semi-public user information may lead to risks such as identity theft, online stalking, and cyber harassment, and may negatively impact users in the future. Privacy concerns are not only one of the most popular cultural values in virtual environments but also the values most closely related to studies on educational virtual communities (Xie et al., 2020).

Therefore, individual privacy concerns are particularly important in the process of user purchase intentions arising from the educational livestreaming platform, because they help to enhance privacy protection, strengthen user psychological ownership, and weaken the impact of psychological ownership to purchase intention process concerns, they are seen as an indispensable lubricant in the virtual environment. Since this is the case, privacy concerns should help reduce the negative effects caused by user experience. Therefore, based on existing studies (Wang and Zhao, 2014; Jiang, 2019), this article will further examine the moderating effect of individual differences in privacy concerns on the relationship between user experience and purchase intention in the context of educational livestreaming platform.

Obviously, it is urgent to investigate the mechanism of “user experience in consumer purchase intention in the context of educational livestreaming platform.” To this end, this article introduces psychological ownership as a mediating variable and privacy concern as a moderating variable from the perspective of user experience, and constructs a theoretical model of the influence mechanism of user purchase intention to explore in depth the influence of user experience on their purchase intention and the difference of the moderating effect of different degrees of privacy concern on the outcome of purchase intention.

## Theoretical Background and Research Hypotheses

### User Experience

In recent years, with the increase of users' demand for experience, scholars' research on user experience has been deepened and the application area of research has been expanded, and user experience has been gradually applied to the field of enterprise marketing (Ogara et al., 2014; Yang et al., 2020). Schmitt (1999) defined user experience as a direct observation and feeling that arise from an individual's personal involvement in an activity, which originates from stimulation of the senses, mind, and heart to induce the perception of individual events, and there are no two experiences that are exactly the same. A good feeling arises in the mind when an individual reaches a certain level of an emotion or feeling under the influence of an experience (Pine and Gilmore, 1999; Yang et al., 2021). Li et al. (2019a) argue that user experience has clear beginning and end times, and that all feelings that a user gets during these times are called user experience. From an enterprise perspective, user experience is considered as the intuitive experience of users during the use of software systems or hardware systems, such as interface design, voice conversion, and text expression. This characterizes the psychological response that an individual gets from contact with an organization (Meyer and Schwager, 2007; Zhao and Ban, 2021).

Educational livestreaming platforms have gathered a large number of users to participate in them because of their superior experience value function, and the marketing value is increasingly prominent, which makes users pay more and more attention to the experience they get in the consumption process (Guo et al., 2021). Therefore, research exploring the marketing of educational livestreaming platforms from the perspective of user experience has also received attention from scholars. Cova et al. (2007), in their study of virtual environments, found that when users' perceived experiences in using a virtual platform meet expectations, they will develop positive attitudes toward that virtual platform and further influence users' perceptions of that virtual platform. Nambisan and Watt (2011) formally proposed that user experience in an online virtual context refers to the full range of feelings that a user acquires in that virtual environment. As an educational livestreaming platform with features such as shopping recommendation, information search, and product purchase, its user experience. includes functionality, information, and product experience. This study combines previous studies (Carlson and Zmud, 1999; Nambisan and Watt, 2011; Ogara et al., 2014) to define user experience in the context of educational livestreaming platform. User experience in the context of educational livestreaming platform is defined as a series of stimulation of the user's mind and heart by the direct observation and reaction when using the educational livestreaming platform to find information, share goodies, communicate, and purchase products, which induces a good feeling about the educational livestreaming platform. User experience is not only memorable for the user but also a strong driver of subsequent behavior.

#### Psychological Ownership

Psychological ownership first emerged in corporate organizations as a “sense of psychological ownership” of the organization by the individual (Pierce et al., 2001). Pierce et al. (2001) argued that employees develop psychological ownership of their organizations, which makes them feel that the target or a part of the target belongs to them and is essentially a sense of possession. First, the core concept of psychological ownership is a sense of ownership of the target, expressed in the emotion or meaning associated with being “mine” or “ours.” Second, psychological ownership reflects the fact that the individual exhibits a concept of self that is closely connected to the target and can even be an extension of the self. Finally, psychological ownership has both cognitive and affective components. Clearly, psychological ownership reflects the individual's awareness, thoughts, and beliefs about the ownership of the target, along with the affective components of pleasure and self-efficacy. Research suggests that psychological ownership can be effectively engendered and enhanced through increased control, greater input, and increased familiarity (Pierce et al., 2001). Regarding the results of psychological ownership, most scholars agree that psychological ownership will trigger positive attitudes and positive improvement in user behavior toward a company or a brand, such as customer loyalty and purchase intention. When users have psychological ownership, they will have a positive relationship with a company and, thus, behave in a way that is beneficial to the company.

Studies have shown that companies motivate employees' sense of efficacy, space, and self-identity in different ways to create psychological ownership, and that the positive attitudes, self-concept, and sense of responsibility shown by employees' psychological ownership lead to positive behaviors toward the company (Lee and Suh, 2015). Further studies by scholars such as Jussila et al. (2015) and Kirk et al. (2018) found that not only employees have psychological ownership of the organization but that users also have psychological ownership of the firm or product. It should be noted that psychological ownership differs from formal ownership in that formal ownership is the possession of real rights such as property rights and control or knowledge of the target that are recognized or protected by law, while psychological ownership only has a sense of control, possession, and identity over the target that is recognized and protected by the individual themselves' virtual rights (Kirk et al., 2018).

Based on the definition of psychological ownership by previous scholars and the characteristics of the educational livestreaming platform, the psychological ownership in this study refers to the user's “psychological possession” of the educational livestreaming platform. It is a state of consciousness in which users feel that the educational livestreaming platform belongs to them, and this state of consciousness makes them have a more positive value judgment and a more positive attitude toward the educational livestreaming platform they are on. This state of consciousness leads to more positive value judgments and behavioral responses to the platform. There are abundant studies on the results of psychological ownership, but fewer studies have introduced psychological ownership into educational livestreaming platform. Therefore, this study introduces psychological ownership into the study of marketing of educational livestreaming platform and explores the mechanism of psychological ownership on educational livestream platform.

### User Experience and Psychological Ownership

Sirgy (2018) stated that the more an individual is invested in the target, the stronger the connection between the self and the target will be and, thus, the easier it will be to feel ownership of the target. Therefore, when users use the educational livestreaming platform, they invest their time and energy into the educational livestreaming platform.The higher the level of commitment, the stronger the connection between the user's perceived self and the educational livestreaming platform. This leads to a sense of belonging and identity to the educational livestreaming platform, and a state that the educational livestreaming platform is “self,” which leads to a sense of psychological possession of the educational livestreaming platform. In other words, this leads to psychological ownership of the educational livestreaming platform by the users. It has been shown that individual psychological ownership arises from three aspects of efficacy, spatiality, and self-identity (Pierce et al., 2001).

In the educational livestreaming platform context, users feel that the information experience is valuable to them, which helps them understand the product information, enhances their sense of control over the product, stimulates the self-efficacy of control, and promotes their psychological ownership. That is, user experience positively affects psychological ownership through a sense of efficacy. In the context of educational livestreaming platform, the interaction between users and users with the same interests not only allows them to fully express and present themselves but also constructs themselves from the support and recognition of others and strengthen their self-identity, and psychological ownership is generated. That is, user experience through self-identity can influence psychological ownership. In the context of educational livestreaming platform, a series of emotions and feelings that users, when a friendly emotion occurs between users and other members of educational livestreaming platform, this emotional attachment will give users a sense of spatial belonging, and once the sense of belonging is satisfied, the user's psychological ownership of educational livestreaming platform will be enhanced, i.e., user experience. In other words, user experience stimulates the occurrence of psychological ownership through a sense of belonging.

In addition, users perceive the commonality or uniqueness between themselves and the educational livestreaming platform, which leads to the desire to become better. This experience not only makes users have a positive attitude toward the educational livestreaming platform but also motivates them to change their existing selves, satisfying their desire to construct themselves better, stimulating a sense of efficacy, and effectively promoting psychological ownership (Zhao and Jing, 2015). Yao et al. (2016) used an empirical research method to examine the effect of user experience on the strength of consumer-brand relationship using psychological ownership as a mediating variable, and found that the better brand experience consumers receive, the higher their psychological ownership. Based on this, this study inferred the following research hypothesis.

Hypothesis 1: user experience in educational livestreaming platform has a positive effect on psychological ownership.

### User Experience and Purchase Intention

The educational livestreaming platform has broken the time and geographical limitations of traditional e-commerce, enabling users to have a greater user experience, and has attracted the attention of scholars and business managers (Brekke et al., 2021; Huang and Xu, 2021). User experience can lead consumers to form positive attitudes toward a brand or a product (Tan, 2019). Scholars have also noticed early on that there are some relationships between user experience and behavioral intention of educational livestreaming platform users. For example, Li et al. (2019b) argued that companies should pay special attention to customers' brand experience and continuously improve their sensory, emotional, thinking, behavioral, and associative experiences so as to further enhance customer brand relationships. Qu and Zhang (2015) then pointed out that the user experience of virtual platforms can directly affect user satisfaction and, ultimately, user consumption intention.

Given the rapid development of information technology and its convenience to users' lives, many consumers no longer passively receive news from companies but rely more on searching for other consumers' opinions on the use of products (Wang, 2021). The social nature of the live education platform itself makes the platform very focused on some product information, as well as good user experience. The positive interaction of users in educational livestreaming platform can enhance their favorable feeling and attention to educational livestreaming platform, so the interactive user experience has a positive effect on purchase intention. In addition, emotion-based user experience is a subjective experience, and a good emotional experience helps to form community satisfaction, which further enhances their purchase intention; association-based user experience helps to create some kind of psychological connection between users and the community, which gives users a kind of satisfaction beyond the product and enhances their positive evaluation of the community, thus driving consumers to form purchase intention (Sun and Chen, 2019). Based on the above discussion, this study proposed hypothesis 2 as follows:

Hypothesis 2: user experience in educational livestreaming platform has a positive effect on purchase intention.

### Psychological Ownership and Purchase Intention

Psychological ownership not only forms a psychological response to it but also influences the subsequent consumption behavior of consumers (Micu and Ashley, 2021). Especially in the face of overcapacity and fierce competition, by introducing customer psychological ownership and exploring its influence role in customer engagement behavior, it can effectively motivate customers to pay attention to the future development of the company and actively participate in various activities of the company, creating more value for the subsequent development of the company and providing certain reference (Zhang et al., 2016). In the field of research related to educational livestreaming platform, researchers on psychological ownership and customer behavior generally agree that the power and attraction generated by the livestreaming platform affect user behavior and believe that psychological ownership has an important value in influencing the motivation of customer behavior (Lu et al., 2021). Gong et al. (2020) conducted a study on how live streaming platforms such as Youtube Live, Twitch, and Periscope use appearance design to enhance sustainable marketing effectiveness. The study addresses the shortcomings of existing studies that rarely discuss the design of live streaming platforms on their users' impulse purchases, and explores the results obtained using multiple linear regressions based on self-determination theory to show that the psychological ownership exhibited by users plays a simultaneous and interlocking mediating role in the relationship between the platform's design and users' impulse purchases.

In the educational livestreaming platform, psychological ownership allows customers to have a higher opinion of the product before purchasing it and to consider it as their own property psychologically, so that they are not only willing to buy it themselves but also to share it with their friends and family and recommend it (Heo et al., 2020). To enhance this marketing effect, educational livestreaming platforms focus on designing pleasant external cues, creating a better psychological atmosphere, and enhancing users' self-efficacy, relying on psychological ownership to enable users' sensory upgrades and promoting better interaction and understanding between products, brands, and users, thus triggering positive attitudes toward the company or brand and positive improvements in purchase intention (Ham and Lee, 2020). This will lead to positive attitudes and positive purchase intentions toward the company or brand (Ham and Lee, 2020). Therefore, in the context of educational livestreaming platform, users develop psychological ownership, which in turn affects purchase intention. Based on this, this study inferred the following research hypothesis:

Hypothesis 3: psychological ownership in educational livestreaming platform has a positive effect on purchase intention.

### Mediating Role of Psychological Ownership

Users' psychological ownership may be an important mediating conductive variable from consumption experience to purchase intention (Meng and He, 2019; Cheng et al., 2020). The stronger the user's sense of experience of a thing, the more deeply and thoroughly he or she can feel it, and in this process, he or she establishes a connection between the self and the thing, enhancing the individual's sense of possession of the target, thus creating a sense of ownership of the target and further promoting consumption behavior. Based on social exchange theory, Cheng et al. (2020) argued that psychological ownership plays a mediating role in the influence of social capital in virtual communities on customers' citizenship behaviors. Meng and He (2019) pointed out the mediating role of psychological ownership between customers' multiple interactions and citizenship behavior tendencies. Scholars Sinclair and Tinson (2017) pointed out that users' knowledge of the platform and familiarity have a positive impact on psychological ownership. In the educational livestreaming platform, users' familiarity and understanding of the platform is enhanced based on their enhanced experience with the platform, which leads to increased sense of control over the platform, and user behavior research has found that control has a positive effect on customers' psychological ownership. Therefore, users' experience in using the educational livestreaming platform has a positive effect on psychological ownership. In addition, Zhu and Liu (2011) reviewed the main theoretical literature on psychological ownership and latest empirical studies, and comprehensively examined the frontier progress of psychological ownership research at home and abroad from three aspects: concepts, antecedent variables and generative mechanisms, and outcome effects, and summarized several important developments in psychological ownership research in recent years. In recent years, psychological ownership research has evolved from focus on individual possession experiences to focus on social interaction processes; from focus on main effects to focus on complex moderating effects; and from focus on Western organizational contexts to focus on multiple forms of organizational contexts in other cultures. According to Zhu and Liu (2011), it can be found that psychological ownership tends to be defined in social interactions, and that ownership beliefs tend to be formed in social interactions.

To sum up, users develop a strong sense of ownership or possession of the educational livestream platform, which will make them psychologically see the educational livestream platform as part of themselves. In this context, they will care about the long-term development of the educational livestream platform and participate more actively in shopping of educational livestream platform, further increasing their purchase intention. Therefore, the user experience of the educational livestream platform will influence their purchase intention through psychological ownership. Based on this, this study inferred the following hypothesis:

Hypothesis 4: psychological ownership in educational livestreaming platform plays a mediating role between user experience and purchase intention.

### Moderating Role of Privacy Concerns

Privacy concerns refer to users' perceptions and concerns about the collection, acquisition, monitoring, and use of personal information, reflecting the subjective feelings of individuals about the corresponding privacy situations (Son and Kim, 2008; Ou and Yuan, 2016; Wang et al., 2021). For a user in a virtual context, privacy concerns are an important prerequisite for making security decisions and an important antecedent variable for privacy behavior. It has been shown that there is a positive relationship between privacy concerns and information security privacy behavior willingness, i.e., one study suggested that privacy concerns are an important trigger for individuals' motivation to engage in protective behavior (Zhao, 2016). By comparing personal information disclosure in the United States and Germany, a positive effect of privacy concerns on users' willingness to protect information security privacy was found (Zhang, 2010). On social media, privacy concerns have a positive effect on users' willingness to set privacy (Luo et al., 2020; Zhao et al., 2021).

Although existing research has found that psychological ownership leads to direct positive feedback from users to the educational livestreaming platform (e.g., Sheehan and Hoy, 2000), growing body of recent research suggests that the effect of psychological ownership on user behavior is inhibited by a number of factors. These inhibiting factors affecting the role of psychological ownership include characteristics of the message (Mothersbaugh et al., 2012), individual user psychological traits (e.g., moderating focus and level of interpretation) (Mosteller and Poddar, 2017), characteristics of interaction management (Taddicken, 2014), and cultural differences (Park et al., 2015). In other words, psychological ownership motivates users to behave positively toward the educational livestreaming platform only if certain conditions are met. Therefore, the effect of psychological ownership on purchase intention is moderated by privacy concerns.

It can be seen that in the context of educational livestreaming platform usage, users with low levels of privacy concerns do not have a significant positive inhibitory effect of privacy concerns on psychological ownership affecting purchase intention relationships. In contrast, users with high levels of privacy concerns have a significant negative moderating effect of privacy concerns on the relationship between psychological ownership and purchase intention, but are more concerned about the negative effects of inclusion compared to users with low levels of concerns. In other words, in the context of educational livestreaming platform use, as the level of users' privacy concerns increases, the effect of users' psychological ownership on purchase intention is consumed. Therefore, this study concludes that the relationship between psychological ownership and purchase intention is positively inhibited by privacy concerns; however, the moderating effect of privacy concerns on the influence of psychological ownership on purchase intention has not yet received sufficient attention and discussion.

Hypothesis 5: privacy concerns in educational livestreaming platform play a negative moderating role between psychological ownership and purchase intention.

**Figure 1:**
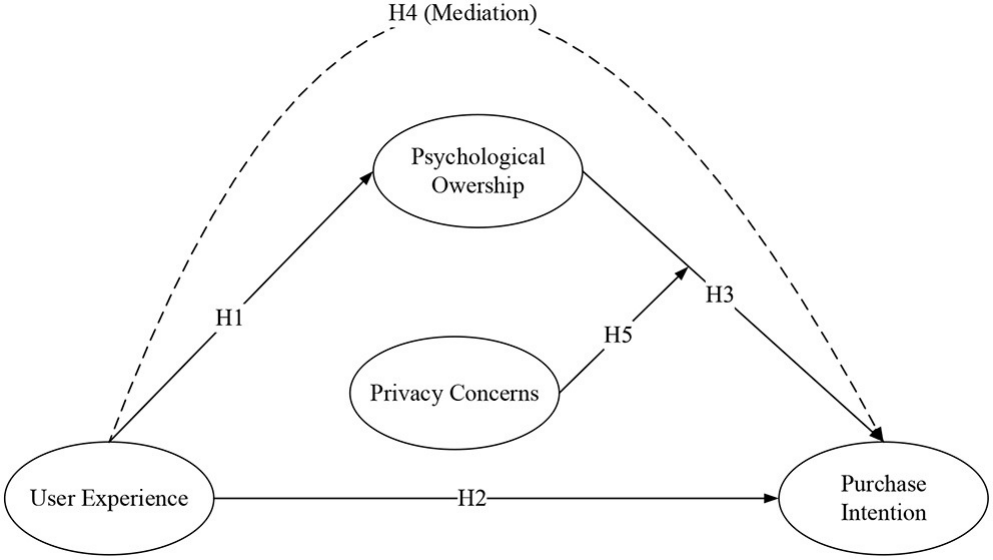
Theoretical model.

In summary, this study proposes a research model as shown in Figure 1.

### Sample and Procedures

To ensure the content validity of the questionnaire, the measurement items were mainly adapted from the previous related literature, and some of the measurement items were modified according to the application contexts of educational livestreaming platform. All the items were rated on a 7-point response scale ranging from 1 (strongly disagree) to 7 (strongly agree). The measurement items are shown in Table 1.

**Table 1:** Variables and measurement items.

**Variables**	**Items**	**Sources**
User experience	UE1. I am very experienced using educational livestreaming platform.	Carlson and Zmud (1999); Ogara et al. (2014)
	UE2. I feel that educational livestreaming platform is easy to use.	
	UE3. I feel competent using educational livestreaming platform.	
	UE4. I understand how to use all of the features of educational livestreaming platform system.	
	UE5. I feel comfortable using educational livestreaming platform.	
Psychological ownership	PO1. I sense that this Himalaya is our educational livestreaming platform.	Van Dyne and Pierce (2004)
	PO2. I feel a very high degree of personal ownership for this Himalaya.	
	PO3. I sense that this is my educational livestreaming platform.	
	PO4. Himalaya is our educational livestreaming platform.	
	PO5. Most of the people that use this Himalaya feel as though they own the educational livestreaming platform.	
Purchase intention	PI1. I would recommend this educational livestreaming platform to my friend.	Wang et al. (2019)
	PI2. I would buy the product or service of the educational livestreaming platform.	
	PI3.There is a probability that I would consider buying the product or service of the educational livestreaming platform.	
Privacy concerns	PC1. I am concerned that my personal information on educational livestreaming platform will have a negative impact on me.	Son and Kim (2008); Tan et al. (2012)
	PC2. I am concerned that my personal information on the educational livestreaming platform may be misused.	
	PC3. I am worried that my personal information on educational livestreaming platform will be used by others.	
	PC4. I am concerned that using the educational livestreaming platform will reveal my private information.	
	PC5. I am concerned about the negative consequences of unknown parties accessing myprivate information on this educational livestreaming platform.	
	PC6. I am concerned that unknown parties have access to my private information on thiseducational livestreaming platform.	

The educational livestreaming platform is currently a popular virtual community product with a relatively large user scale and rich product types. Considering the representativeness of the sample collection, users of the most popular Himalaya in China were selected as the research subjects in this study. Himalaya is a mobile client launched in March 2013, more than 2 years mobile user scale has exceeded 200 million, becoming the fastest growing and largest online mobile educational livestreaming platform in China. Two rounds of high financing were completed within 2014 to further lead the Chinese educational livestreaming platform in China. With Himalaya, consumers can upload their work using their fingertips, create a personal live platform dedicated to them, and continue to grow and accumulate fans and stay connected to them. This shows that Himalaya is representative and typical as an educational livestreaming platform.

The data collection method was mainly used to obtain the sample data using the web platform provided by the web company “Questionnaire Star” to form an electronic questionnaire, and then the web link of the electronic questionnaire was published in each browser community. In order to improve the efficiency of the questionnaire collection, each user who fills out the questionnaire is given a reward of 5 yuan. The questionnaire survey was conducted from November 2021 to March 2022, and 472 questionnaires were obtained by this method, excluding invalid questionnaires there, forming 372 valid questionnaires. The sample information is shown in Table 2.

**Table 2:** Descriptive statistical analysis.

**Variables**	**Item**	**Frequency**	**%**	**Cumulative %**
Gender	Male	121	32.5	32.5
	Female	251	67.5	100.0
Age (year)	20 or less	44	11.8	11.8
	21~30	269	72.3	84.1
	31~40	17	4.6	88.7
	41~50	22	5.9	94.6
	51 or above	20	5.4	100.0
Marriage	Married	57	15.3	15.3
	Unmarried	315	85.7	100.0
Education level	High school and below	22	5.9	5.9
	College	49	13.2	19.1
	Undergraduate	262	70.4	89.5
	Master's degree and above	39	10.5	100.0
Consumption (RMB)	Below 2,000	148	39.8	39.8
	2,000~3,999	99	26.6	66.4
	4,000~5,999	53	14.2	80.6
	6,000 or more	72	19.4	100.0
Continuous use time (year)	<1	148	39.8	39.8
	1~2	134	36.0	75.8
	Over 3	90	24.2	100.0

## Data Analyses

### Measurement Model Analysis

This study evaluates and revises the Confirmatory Factor Analysis (CFA) measurement model based on a two-stage model (Kline, 2011). Currently, academics generally agree with the approach of Anderson and Gerbing, 1998. That is, CFA should report factor loadings, Cronbach's alpha, composite reliability (CR), and average variance extracted (AVE) for all variables, and only after these metrics pass the test can structural models be evaluated (i.e., factor loading > 0.5, Cronbach's alpha > 0.7, CR > 0.6, and AVE > 0.5) (Fornell et al., 1981; Nunnally and Bernstein, 1994), then the measurement model has good convergent validity. Table 3 reports the CFA of the measurement models. Among them, factor loadings of all dimensions are between 0.64 and 0.817, Cronbach's alpha is between 0.828 and 0.865, CR is between 0.832 and 0.868, and AVE is between 0.525 and 0.622, indicating that each construct has good convergence validity.

**Table 3:** Confirmatory factor analysis.

**Variables**	**Items**	**Factor loadings**	**Cronbach's alpha**	**CR**	**AVE**
User experience	UE1	0.734	0.854	0.854	0.541
	UE2	0.817			
	UE3	0.707			
	UE4	0.718			
	UE5	0.694			
Psychological ownership	PO1	0.687	0.847	0.851	0.534
	PO2	0.775			
	PO3	0.814			
	PO4	0.664			
	PO5	0.704			
Purchase intention	PI1	0.768	0.828	0.832	0.622
	PI2	0.801			
	PI3	0.797			
Privacy concerns	PC1	0.640	0.865	0.868	0.525
	PC2	0.640			
	PC3	0.807			
	PC4	0.757			
	PC5	0.760			
	PC6	0.726			

The results of the discriminant validity analysis are shown in Table 4. Discriminant validity is a measure to test whether any two variables in a theoretical model are identical to each other. In this study, discriminant validity was analyzed using a method commonly used in structural equation modeling, namely, the interval method of confidence values (Torkzadeh et al., 2003). The confidence interval method is used to confirm the confidence interval of the correlation coefficient between variables. If it fails to include “1,” then it is completely correlated, indicating that the facets have different validity. As Hancock and Nevitt (1999) suggested, a bootstrap test was conducted in this study, and 95% confidence interval of the correlation coefficient does not involve 1, which shows good discriminant validity between all the variables. Therefore, the measurement model has good discriminat validity.

**Table 4:** Discriminant validity.

**Parameter**	**Correlation coefficients**	**Bias-corrected 95%**	
		**Lower**	**Upper**
UE < ->PO	0.607	0.482	0.710
UE < ->PI	0.607	0.465	0.715
UE < ->PC	0.726	0.655	0.795
PO < ->PI	0.715	0.506	0.878
PO < ->PC	0.726	0.559	0.857
PI < ->PC	0.725	0.591	0.821

*UE, user experience; PO, psychological ownership; PI, purchase intention; PC, privacy concerns*.

### Stuctural Model Analysis

This study used a wide range of methods used in previous structural equation modeling studies to analyze structural model fit. That is, nine goodness-of-fit indicators were analyzed to determine whether the study model has a good fit (Jackson et al., 2009). In SEM analysis, if the sample size is larger than 200, it will cause chi-square to inflate, leading to decreased model fit (Bollen and Stine, 1992). This study used Bollen-Stine Bootstrap to corrected SEM chi-square. After Bollen-Stine bootstrapping correction, the model indices fit all the suggested criteria (refer to Table 5). Therefore, the structural model of this study has a good model fit.

**Table 5:** Model fit criteria.

**Model fit**	**Criteria**	**Model fit of research model**
MLχ^2^	The small the better	70.707
DF	The large the better	62
Normed Chi-sqr (χ^2^/DF)	1 < χ^2^/DF <3	1.140
RMSEA	<0.08	0.019
SRMR	<0.08	0.028
TLI (NNFI)	>0.9	0.996
CFI	>0.9	0.996
GFI	>0.9	0.972
AGFI	>0.9	0.947

### Hypothetical Testing

The regression coefficients are shown in Table 6. User experience (UE) (β = 0.605, *p* < 0.001) has a positive and significant impact on psychological ownership (PO). Therefore, hypothesis 1 is supported. User experience (UE) (β = 0.269, *p* < 0.001) has a positive and significant impact on purchase intention (PI). Therefore, hypothesis 2 is supported. Psychological ownership (PO) (β = 0.553, *p* < 0.001) has a positive and significant impact on purchase intention (PI). Therefore, hypothesis 3 is supported.

**Table 6:** Path coefficient and hypothesis testing.

**Hypothesis**	**Unstd. coefficient**	**S.E**.	***z***	**Std. coefficient**	***p***
UE->PO	0.528	0.060	8.863	0.605	***
UE->PI	0.243	0.060	4.078	0.269	***
PO->PI	0.573	0.078	7.391	0.553	***

*** *p < 0.001; UE, user experience; PO, psychological ownership; PI, purchase intention; PC, privacy concerns*.

The results of the intermediary effect analysis are shown in Table 7. In this study, structural equation modeling was conducted to analyze the mediating effect, and the standard error of the mediating effect was first estimated using the Bootstrap estimation technique, and then the significant level of the mediating effect was further calculated. According to Hayes (2009), a mediating effect is indicated if “0” does not include the 95% confidence interval of bias-corrected confidence interval range, the z-value is >1.96, and the *p*-value is < 0.05.

**Table 7:** Analysis of mediation effect.

**Indirect effect**	**Path coefficient (β)**	**Product of coefficients**			**Bootstrap**	
					**Bias-corrected 95%**	
		**SE**	***Z***	***p***	**Lower**	**Upper**
Total effect: UE → PI	0.546	0.081	6.741	0.005	0.376	0.694
Indirect effect: UE → PO → PI	0.303	0.113	2.681	0.003	0.132	0.560
Direct effect:UE → PI	0.243	0.120	2.025	0.054	−0.005	0.475

*UE, user experience; PO, psychological ownership; PI, purchase intention; PC, privacy concerns*.

The total effect of user experience on purchase intention is 0.546. At the 95% confidence level, “0” does not include the bias-corrected 95% confidence interval range, the z-value is >1.96, and the *p*-value is <0.05. Therefore, there is a total effect that exists. The indirect effect is 0.303, “0” does not include the bias-corrected 95% confidence interval range, the z-value is >1.96, and the *p*-value is <0.05. Therefore, there is an indirect effect. The direct effect is 0.243, “0” includes the bias-corrected 95% confidence interval range, the Z-value is >1.96, and the *p*-value is not <0.05. Therefore, no direct effect exists. Therefore, hypothesis 4 is established and is a partial mediation.

The moderating effects are reported in Table 8. In this study, privacy concern (PC) is the moderating variable. The results of the structural equation modeling showed that the moderator effect of psychological ownership (PO) × privacy concerns (PC) on purchase intention (PI) is −0.073 (*z* = |−3.04| > 1.96, *p*-value <0.01), implying the presence of a negative moderating effect of privacy concern (PC) on the relationship between psychological ownership (PO) and purchase intention (PI). Specifically, the slope of psychological ownership (PO) on purchase intention (PI) increases negatively by −0.073 units for each 1-unit decrease in the moderating variable privacy concern (PC). That is, privacy concern (PC) has a positive moderating effect. Therefore, hypothesis 5 is verified.

**Table 8:** Analysis of moderating effect.

**Dependent variable (DV)**	**Independent variable (IV)**	**Path Coefficient (β)**	**S.E**.	***z***	***p***
Purchase intention (PI)	Psychological ownership (PO)	0.492	0.078	6.344	***
	Privacy concerns (PC)	0.406	0.093	4.385	***
	Psychological ownership (PO) × privacy concerns (PC)	−0.073	0.024	−3.040	**

** *p < 0.01*,

*** *p < 0.001*.

## Research Results and Discussion

### Discussion

First, the results of the data analysis confirm the role of user experience on purchase intention. The findings are consistent with those of Tan (2019) and Huang and Xu (2021), indicating that the better the user experience in using the educational livestreaming platform, the stronger the purchase intention. Specifically, the experience that users feel in the process of shopping of educational livestreaming platform is one of the important antecedents of their purchase intention. In the context of educational livestreaming platform supported by the Internet, users focus on the experience of information value, communication interaction, and emotional attachment between educational livestream platform and themselves. When individuals perceive a strong user experience in educational livestreaming platform, they are likely to have purchase intention. Clearly, user experience can positively and significantly influence specific online purchase intention. In the context of educational livestream platform usage, a good user experience helps individuals to obtain useful information and facilitates individuals to have a more comprehensive understanding of educational livestream platform goods. Therefore, when users reap good user experience, it will promote purchase intention. Good user experience can prompt users to establish a strong bond with educational livestream platform and consciously participate in activities such as product sharing and shopping communication on educational livestream platform, thus stimulating the generation of purchase intention. This stimulates purchase intention.

Second, the results of the structural equation modeling indicate that user experience has a significant effect on psychological ownership. The findings confirm the positive effect of user experience on psychological ownership, and the findings are consistent with the results of existing studies such as Zhao and Jing (2015) and Yao et al. (2016), which indicate that the better the user's experience on the educational livestreaming platform, the better the experience they get and the stronger their psychological ownership. Educational livestream platform as a new e-commerce platform, by providing certain functions to meet the needs of the majority of users, is actually to provide users with a certain external environment stimulation, users under the stimulation of this external environment, combined with their own shopping needs, and constantly deepen their understanding of educational livestream platform. The user is immersed in the promotion of the educational livestreaming platform content. This positive psychological state enhances the relationship between the user and the educational livestream platform, prompting the user to develop a cognitive-emotional and conscious state that the educational livestream platform belongs to him or her, which in turn stimulates a sense of possession, i.e., psychological ownership. At the same time, the educational livestreaming platform uses big data technology to understand and analyze users' past browsing behavior, more accurately recommend products of interest to users, and recommend some educational livestreaming platform experts to share their experience in related products. These user experiences increase the influence on users and enhance their expectation to become better. As a result, user experience is enhanced, and user purchase intention is promoted.

Third, the results of the structural equation modeling indicate that psychological ownership has a significant effect on purchase intention. The findings confirm the hypothesis that psychological ownership affects purchase intention and are consistent with Ham and Lee (2020) logical reasoning, suggesting that the more psychological ownership users generate on the educational livestreaming platform ownership of the educational livestreaming platform, the more likely they are to generate purchase intention. The reason may be that individuals become more and more connected with other members under the stimulation of the experience, thus invariably establishing a stronger psychological ownership, which leads to a sense of satisfaction, attachment, and belonging to the educational livestream platform, and the recognition of the educational livestreaming platform is enhanced, which in turn stimulates their purchase intention. In addition, the pleasure and satisfaction brought by the educational livestream platform helps to satisfy the individual's sense of possession, which in turn leads to the user's purchase intention. In other words, educational livestreaming platform realizes that psychological ownership plays an important role. It is not only an important bridge for communication and interaction between users and educational livestreaming platform but also effectively helps educational livestreaming platform to grasp the psychological activities of users. Therefore, psychological ownership can help educational livestreaming platforms to develop targeted and reasonable marketing strategies and suggestions and then improve their performance.

Fourth, the results of the data analysis indicate that psychological ownership has a significant effect in mediating the effect of user experience on purchase intention. The results of the study confirm the hypothesis that psychological ownership affects purchase intention and are consistent with the logical reasoning of the existing literature. This suggests that the better the user experience in the educational livestream platform, the more likely the user is to develop psychological ownership of the educational livestream platform and the more likely the user is to develop purchase intention. The reason for this situation may be that in the process of using the educational livestreaming platform, the individual gets not only fun but also a sense of psychological satisfaction and pleasure while gaining user experience. In this process, the stronger the user experience, the more the individual feels that the educational livestreaming platform or a part of the educational livestreaming platform belongs to the individual, and the stronger the sense of psychological possession of the educational livestreaming platform. In this process, the user develops psychological ownership and tends to associate the self with the educational livestreaming platform. Users strengthen their knowledge of the educational livestreaming platform and enhance their love for the educational livestreaming platform through continuous access to information and interaction. In this context, users gradually develop stronger psychological ownership, which in turn increases and stimulates their purchase intention.

Fifth, the results of the structural equation modeling indicate that privacy concerns have a significant negative moderating effect on the relationship between psychological ownership and purchase intention. The findings confirm the moderating role of privacy concerns between psychological ownership and purchase intention, and the results are consistent with Yin and Zhang's (2020) logical deduction results. This indicates that the higher the privacy concerns of users on the educational livestreaming platform, the stronger the negative effect of psychological ownership on purchase intention. Although the value created by the educational livestreaming platform can lead individuals to have purchase intention. However, privacy concerns counteract the positive effects of psychological ownership in this process, which in turn counteracts to some extent the facilitative effect of psychological ownership on purchase intention. Thus, as a reflection of the individual's anxiety and concern in the educational livestreaming platform, privacy concerns stimulate a strong sense of responsibility and enhance self-protection. This inevitably hinders individuals' purchase intention, as well as the positive influence of psychological ownership on purchase intention. In addition, the educational livestreaming platform organizes offline gatherings of learners at the right time as a way to enhance the impact of psychological ownership on individual purchase intention, which is a major reason to mitigate the negative moderating effect of privacy concerns.Therefore, privacy concerns negatively moderate the relationship between psychological ownership and purchase intention in educational livestreaming platform.

### Practical Implications

First is enhancing the user experience of individuals in the educational livestream platform. To strengthen the regulatory mechanism in the product content and provide users with reliable and valuable product information, the educational livestreaming platform can place the most valuable posts or those with the highest number of user comments on the product display page to help users understand the product information more accurately and comprehensively. At the same time, the educational livestreaming platform should also delete or filter posts with extreme emotions or unreliable sources in order to improve the quality of information in the platform to achieve consumer satisfaction, which will lead to purchase intention.

In addition, users of the educational livestreaming platform are mainly young people who are pursuing mavericks and have higher and higher requirements for personalization of products and services display. The educational livestreaming platform should design the website from enhancing the uniqueness of consumers, so that consumers can feel a more authentic shopping platform. At present, powerful information technology provides technical support for online platform, traditional text information or simple picture display is no longer enough to impress consumers, and 3D display, panoramic mode, and other new ways are emerging day by day. Therefore, the educational livestreaming platform can create the most suitable display method for this product, so that consumers can have an immersive feeling when viewing the educational livestreaming platform, which will increase their curiosity about the educational livestreaming platform and enhance purchase intention. The educational livestreaming platform should take various measures to promote the interactive experience among consumers. For example, the platform takes the initiative to throw out the topic discussion and make different reward policies for members who participate in the discussion, so as to attract consumers to actively participate in the platform discussion and express their views, and enhance user experience by stimulating users' interest.

Second is stimulating consumers' psychological ownership of educational livestreaming platform. The research results show that psychological ownership can effectively promote the purchase intention of educational livestreaming platform users, and that psychological ownership of educational livestreaming platform users needs external stimulation to be generated. Ownership requires external stimuli to be generated. Therefore, educational livestreaming platform should take some external measures to stimulate users' psychological ownership to increase consumers' purchase intention. First of all, the educational livestreaming platform should pay attention to the impact of users' self-efficacy on their psychology. For example, the educational livestreaming platform can improve users' skills of using the educational livestreaming platform while increasing users' understanding of the community through online teaching and other means to stimulate users' self-efficacy of control, which in turn promotes users' desire for control and ownership of the community. Then, the educational livestreaming platform should create a warm and pleasant feeling for its members as much as possible. For example, the layout and background music of the educational livestreaming platform create a warm feeling as much as possible to give consumers a sense of intimacy, which in turn leads to a sense of dependence, belonging, and, thus, psychological ownership. Finally, educational livestreaming platform should try to understand the needs of different types of users for products and services, make corresponding policy changes according to the types of users, and promote purchase intention. In addition, educational livestreaming platform should make full use of big data technology to understand and analyze users' past browsing behavior, so as to more accurately recommend products that users are interested in, and recommend to them some experience posts shared by educational livestreaming platform experts on related products, etc. At the same time, it should increase the infectious power to the users, enhance their expectation to become better, improve the associated experience, and promote the purchase intention of the users.

Third is care for the core interests of community users and giving them a greater sense of access. The study found that they have different levels of requirements in terms of how much they perceive the educational livestreaming platform caring about their needs or welfare and how much they value their contributions. The higher the privacy concerns, the more credible the educational livestreaming platform is for consumers, the more reliable they perceive the product information, and the more effective it is in reducing the uncertainties arising from the purchase decision process, which reduces the shopping risk and thus promotes consumers' purchase intention. Therefore, it is necessary to propose a strategy for cultivating high privacy concerns from the perspective of educational livestreaming platform. Educational livestreaming platform managers should change the traditional marketing concept, proactively establish the awareness of providing support for educational livestreaming platform consumers, make full use of modern Internet technology for data mining and analysis, effectively differentiate educational livestreaming platform users according to their data mining and analysis by making full use of modern Internet technologies, effectively differentiate educational livestreaming platform users according to the operation process and participation needs of the educational livestreaming platform, and develop supportive policies for different user groups. For example, for new users of educational livestreaming platform, they can develop activities to encourage new users' purchase behavior by giving them a commemorative gift bag or coupons for their first purchase of educational livestreaming platform products, so as to stimulate new users' purchase intention. In addition, consumers should be able to perceive how much the educational livestreaming platform values their contributions. For consumers who can bring benefits to the platform, such as those willing to share goodies and recommend products or services of the educational livestreaming platform in their circle of friends, certain incentive policies should be formulated and corresponding rewards should be given, such as awarding medals and cashback, to guide consumers to pay attention to the future development of the educational livestreaming platform, thus promoting the purchase intention.

### Research Limitations and Future Research Directions

The present study may have some limitations, and further research can be conducted in the future in the following two aspects. First, in this study, users of the educational livestreaming platform “Himalaya” were selected as the target population, and the sample selection was limited. Future studies may consider selecting more users of other educational livestreaming platforms for validation, in order to enhance the generalizability of the study results. Second, this study focused on the influence of user experience on purchase intention in the context of educational livestreaming platform. The results of this study vary depending on the mediating and moderating variables, and subsequent studies can examine other potential variables, such as personal characteristics and product type, to further enrich the intermediate mechanism of user experience on purchase intention.

## Data Availability Statement

The original contributions presented in the study are included in the article/supplementary material, further inquiries can be directed to the corresponding authors.

## Author Contributions

HZ: conceptualization. YZ: formal analysis and investigation. HZ and YZ: writing (original draft) and writing (review and editing). All the authors have read and agreed to the published version of the manuscript.

## Conflict of Interest

The authors declare that the research was conducted in the absence of any commercial or financial relationships that could be construed as a potential conflict of interest.

## Publisher's Note

All claims expressed in this article are solely those of the authors and do not necessarily represent those of their affiliated organizations, or those of the publisher, the editors and the reviewers. Any product that may be evaluated in this article, or claim that may be made by its manufacturer, is not guaranteed or endorsed by the publisher.
